# PnpProbs: a better multiple sequence alignment tool by better handling of guide trees

**DOI:** 10.1186/s12859-016-1121-7

**Published:** 2016-08-31

**Authors:** Yongtao Ye, Tak-Wah Lam, Hing-Fung Ting

**Affiliations:** HKU-BGI Bioinformatics Algorithms & Core Technology Research Lab, Computer Science Department, University of Hong Kong, Hong Kong, China

**Keywords:** Multiple sequence alignment, Guide trees, Phylogenetic trees

## Abstract

**Background:**

This paper describes a new MSA tool called PnpProbs, which constructs better multiple sequence alignments by better handling of guide trees. It classifies sequences into two types: normally related and distantly related. For normally related sequences, it uses an adaptive approach to construct the guide tree needed for progressive alignment; it first estimates the input’s discrepancy by computing the standard deviation of their percent identities, and based on this estimate, it chooses the better method to construct the guide tree. For distantly related sequences, PnpProbs abandons the guide tree and uses instead some non-progressive alignment method to generate the alignment.

**Results:**

To evaluate PnpProbs, we have compared it with thirteen other popular MSA tools, and PnpProbs has the best alignment scores in all but one test. We have also used it for phylogenetic analysis, and found that the phylogenetic trees constructed from PnpProbs’ alignments are closest to the model trees.

**Conclusions:**

By combining the strength of the progressive and non-progressive alignment methods, we have developed an MSA tool called PnpProbs. We have compared PnpProbs with thirteen other popular MSA tools and our results showed that our tool usually constructed the best alignments.

## Background

Constructing multiple sequence alignments (MSA) is an important problem in Bioinformatics. For sequences with sufficiently high similarity, there exist many MSA tools that can produce good alignments, but for sequences with similarity below 30 %, no tools have satisfactory performance. However, these sequences are also of great interest to biologists because even though they have low similarity, many of them have similar secondary and tertiary structures. This paper introduces a new software tool PnpProbs. It can construct significantly better alignments for sequences with low similarity, and it also improves the alignments for general input.

PnpProbs is based on an adaptive approach we proposed in [1], in which we observed that sequences having different similarities have different characteristics and structural properties, and by using some reliable measure to estimate the similarity of the input (we do not know the true similarity because we do not have the correct alignments), we may exploit the corresponding properties to help generate better alignments. To study the feasibility of this idea, we have modified the open source code of MSAProbs [2] and developed a new adaptive MSA tool called GLProbs. Roughly speaking, both tools construct the alignments in the following three stages:

- Determine the substitution scores for pairwise sequences based on some pair-Hidden Markov model(s), and then refine the scores to make them consistent with all input sequences.
- Construct a guide tree and based on it align the input sequences progressively to generate the multiple sequence alignment.
- Refine the alignment given by Stage (2) to a better alignment for the final output.

The major difference between MSAProbs and GLProbs is in the first stage: MSAProbs uses a single model to determine the substitution scores, while GLProbs determines the scores adaptively. GLProbs first estimates the similarity of the input sequences by computing its average PID (percent identity), which is defined as follows: the PID of two sequences is the percentage of identical columns in their optimal (pairwise) alignment, and the average PID of a sequence family is the average of the PIDs of every pair of sequences in the family. If the input’s average PID is high, GLProbs uses the global pair-Hidden Markov model (pair-HMM) to determine the scores; otherwise, it uses some local pair-HMMs.

We have made thorough comparisons between GLProbs and a dozen other leading MSA tools, and GLProbs had the highest accuracy in many of the comparisons (see [1] for more details of our evaluation of GLProbs).

In this paper, we have some ideas for improving GLProbs, and we implement them by developing the alignment tool PnpProbs. We have tested PnpProbs extensively on three benchmark databases BAliBASE [3], OXBench [4], and SABmark [5], and in Section “Benchmark comparison”, we compare its performance with 13 leading multiple sequence alignment tools, including 10 using the progressive method: ClustalW [6], Clustal *Ω* [7], T-Coffee [8], MAFFT [9], MUSCLE [10], ProbCons [11], CONTRAlign [12], Probalign [13], MSAProbs [2], GLProbs, and 3 using the non-progressive method: Align-m [14], PicXAA [15], and DIALIGN-PFAM [16]. PnpProbs’ performance is significant better, specially for distantly related sequences. For example, for families of sequences in OXBench with similarity from 0 to 20 %, PnpProbs achieved an improvement (in TC score) over ClustalW by 36.5 %, over PicXAA by 12.9 % and GLProbs by 8.4 %.

We have also evaluated the performance of PnpProbs on phylogenetic inferencing over two benchmarks, namely Yule-Harding tree simulated data [17] and SABmark empirical data [5]. In Section “Phylogenetic analysis”, we compare PnpProbs with five other MSA tools, namely GLProbs, MSAProbs, PicXAA, MUSCLE and ClustalW, and our results showed that the phylogenetic trees generated from the outputs of PnpProbs are closer to the model phylogenetic trees than those constructed from the five other MSA tools.

For verification of our results, all test data can be accessed from [17, 18], and PnpProbs can be downloaded via the link https://github.com/ytye/PnpProbs.

## Ideas for improving GLProbs

We observe some new structural properties and believe that by exploiting them we can further improve GLProbs’ accuracy in general, and improve its accuracy significantly for sequences with low similarity. We focus on improving the second stage of GLProbs. Based on the substitution scores given in Stage 1, this stage determines a guide tree, which is supposed to capture the phylogeny relationship of input sequences. Then, it generates an MSA by performing profile-to-profile alignment according to the order suggested by the guide tree. Unlike GLProbs, we will use an adaptive approach to construct the guide trees. We classify the input sequences into two types: (i) *distantly related sequences*, whose similarities (or more precisely, average PID) are smaller than some threshold (as suggested by our study in Section “Non-progressive alignment for distantly related sequences”, we set it to be 18 %), and (ii) *normally related sequences*, whose similarities are no smaller than the threshold. PnpProbs handles these two types of sequences differently.

**Fig. 1:**
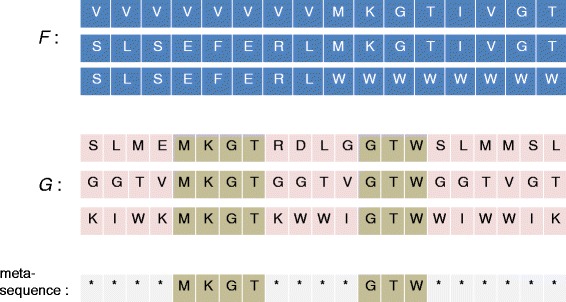
Some structure property that we use for handling normally related sequences

For normally related sequences, we exploit some structure property for better guide tree generation. To explain, we show in Fig. 1 two protein families, *F* and *G*, whose average PID’s are both 0.33, but their structures are quite different. In particular, each sequence of *G* has two regions (which we have highlighted in brown colour) over which the sequences are identical, and the sequences are totally different elsewhere. We note that similar structures can be found in real protein families, especially from those with similarity around 20–30 %:

(*†*) Their sequences have a number of conserved regions over which the sequences are very similar, and the sequences are very different elsewhere.

We have two observations about this structure:

(i) The average PID cannot help us discover (*†*), but the standard deviation can. As shown in Fig. 1, while *F* and *G* have the same average PIDs, the standard deviation of their PID’s are quite different: for *F*, the PIDs of its sequence pairs are 0.5 (1st and 2nd sequences), 0.5 (2nd and 3rd), and 0 (1st and 3rd), and their standard deviation is significantly greater than 0, and for *G*, the PIDs of its sequence pairs are all equal to 0.33 and their standard deviation is 0. This is not surprising because the sequences in *G* are identical over the two conserved regions, and are totally different elsewhere. In general, if a family has small standard deviation of PID, it may have structure (*†*).
(ii) When aligning a family *G* with structure (*†*) to some other family *F*, we should aim at finding alignment that is good mainly over *G*’s conserved regions, because *G*’s sequences are quite different elsewhere and even biologists may not know how to align the sequences correctly over there. Furthermore, since *G*’s sequences are very similar over the conserved regions, having a good alignment (over the conserved regions) for one sequence of *G* will essentially give us good alignments for all the others. This suggests that when aligning *G* to *F*, we may proceed as if we were aligning a single (meta)-sequence to *F* (or more concretely, assume that *G* has only one single sequence).

Observation (i) motivates the following strategy to determine whether a family has structure (*†*): If the standard deviation of the PIDs of the family is sufficiently small, we bet that it has structure (*†*). For ease of reference, we will say that such family has *low PID discrepancy*, or simply low discrepancy.

Observation (ii) motivates us to try a guide tree construction method different from GLprobs’ when handling families with low discrepancy. Note that the method UPGMA [19] is used in GLProbs to construct guide trees. The method iteratively merges clusters of sequences into larger clusters, and the two closest pair of clusters are chosen and merged in each iteration. The distance *d*_*ℓk*_ between two clusters *C*_*ℓ*_ and *C*_*k*_, where *C*_*k*_ is obtained after merging the clusters *C*_*i*_ and *C*_*j*_, is

1 $$  d_{\ell k} = \frac{|C_{i}|}{|C_{i}|+|C_{j}|} d_{\ell i}+\frac{|C_{j}|}{|C_{i}|+|C_{j}|}d_{\ell j}.  $$

In this paper, we try another guide tree construction method for families with low discrepancy; we will use the WPGMA method [19], which is the same as the UPGMA method, but it uses the following definition of distance:

2 $$  d_{\ell k} = \frac{1}{2}d_{\ell i} + \frac{1}{2}d_{\ell j}.  $$

Note that (2) is equal to (1) when |*C*_*i*_|=|*C*_*j*_|=1, or when both *C*_*i*_ and *C*_*j*_ can be regarded as containing only one single sequence (meta-sequence), as suggested by Observation (ii) for families with low discrepancy.

For distantly related sequences, they are only similar at some local domains or motifs, and these homologous regions may be rather small and are hidden in some long divergent regions. This causes troubles for the progressive alignment method, which is based on global pairwise alignments to merge and align iteratively clusters of sequences together to construct the MSA, and the order of merging depends solely on the guide tree. By insisting global alignments for inputs that have only local similarity, the progressive method may introduce, even in the early stage of execution, many mis-aligned columns and other mistakes, and these early mistakes cannot be corrected and may be propagated [20] and create more mistakes. To improve the alignment quality for distantly related sequences, we forget about the progressive methods and instead, we try non-progressive ones.

There exist many non-progressive MSA methods. For example, the non-progressive sequence annealing technique described in [21, 22] combines successively confident alignable regions to build up the multiple alignment; the most similar segments (even in small size) will be aligned first in order to preserve those conserved motifs or domains.

We use this sequence annealing technique to handle input of distantly related sequences. Recall that in Stage 1, we have used the adaptive method to determine substitution scores. During the process, we have also found, for every pair of sequences *x* and *y* in the family, and every 1≤*i*≤|*x*| and 1≤*j*≤|*y*|, the probability Pr(*x*_*i*_,*y*_*j*_) of aligning the *i*th character of *x* and the *j*th character of *y* in the best alignment. To construct an MSA for distantly related sequences, we will first sort all the character pairs (*x*_*i*_,*y*_*j*_) in descending order of Pr(*x*_*i*_,*y*_*j*_). Then, starting from the first character pair in the sort list, which has the highest probability of being aligned at the same column, we follow the character pairs in the list and try to insert each pair to the alignment (or more precisely, make the two characters in the pair aligned at the same column) one by one. However, we will actually make the insertion only if the alignment is still consistent after the insertion.

For checking of consistency, we will maintain a collection of “same-column” sets, which contains all the characters that we have determined that they should be aligned at the same column. We will keep track of these sets using a graph, in which its nodes are the sets, and for any two same-column sets *S* and *S*^′^, we have a direct edge (*S*,*S*^′^) in the graph if the column for *S* must precede that of *S*^′^ in the alignment (e.g., when *S* contains the 10th character of sequence *x* and *S*^′^ contains the 20th of *x*). When we insert a pair (*x*_*i*_,*y*_*j*_) to the alignment, we will update the graph by either

(i) introducing a new same-column set (when both *x*_*i*_ and *y*_*j*_ are not currently in any same-column set), or
(ii) adding either *x*_*i*_ or *y*_*j*_ in some existing same-column set (e.g., if *y*_*j*_ is already in some *S*, then we need to add *x*_*i*_ to *S* after inserting (*x*_*i*_,*y*_*j*_)), or
(iii) merge two same-column sets (e.g., if *x*_*i*_ is already in *S* and *y*_*j*_ in *S*^′^, then after inserting (*x*_*i*_,*y*_*j*_) we need to merge *S* and *S*^′^ together).

We also need to update the edge set of the graph to reflect the changes. Note that we will not actually make the insertion unless the updated graph is still acyclic, which means that the column constraints are still consistency. When we have finished processing all the character pairs in the sorted list, we topological-sort the graph to get a skeleton of the MSA. We obtain the final MSA by adding to it those characters not in the skeleton. See [16, 21–23] for more details.

## Methods

### Construct better guide trees for normally related sequences

**Fig. 2:**
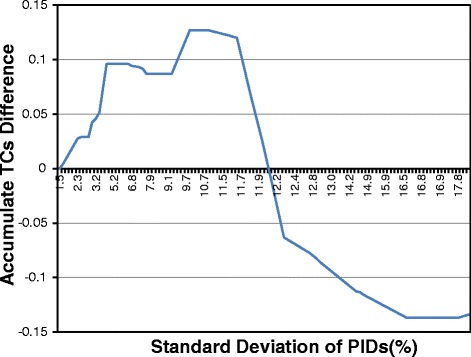
Accumulated TC score difference between GLProbs-WPGMA and GLProbs-UPGMA
- We put a point (*h*,*k*) on the curve if $k =\sum _{i \leq h}\Delta _{i}$, i.e., the accumulated differences up to the group with *σ*(PID) =*h* is *k*.

PnpProbs uses an adaptive approach to generating guide trees for normally related sequences. As mentioned in Section “Ideas for improving GLProbs”, we have two methods, the UPGMA and the WPGMA method to construct guide trees. To study which methods is better, we have modified GLProbs such that it uses the WPGMA method to construct guide trees. For ease of reference, we use GLProbs-UPGMA to refer the original GLProbs, and GLProb-WPGMA to refer the modified one. We used both tools to align the normally related families in SABmark, OXBench and BALiBASE, and compute the TC scores of the resulting alignments, which is one of the most commonly used performance measure for evaluating multiple sequence alignments; the higher the scores, the better. Figure 2 shows the accumulated differences of their TC scores. To explain the figure, let us denote by *σ*_*G*_(PID) the standard deviation of the PIDs over all pairs of sequences in family *G* (we will drop the subscript *G* when there is no confusion). The curve in the figure is constructed as follows.

- We first classify the input families according to their *σ*(PID)s, and for each group *i*, i.e., the group with *σ*(PID) =*i*, we compute the average TC scores $\overline {TC}_{\tt WPGMA}$ and $\overline {TC}_{\tt UPGMA}$ over the alignments returned by GLProbs-WPGMA and GLProbs-UPGMA for the families in this group, respectively. Then, we compute $\Delta _{i} = \overline {TC}_{\tt WPGMA} - \overline {TC}_{\tt UPGMA}$.

Note that if the curve is increasing at (*h*,*k*), we have *Δ*_*h*_>0 and GLProbs-WPGMA is doing better than GLProb-UPGMA. As shown in Fig. 2, the accumulated differences is mainly increasing until *σ*(PID) reaches around 11.5 %, and hence GLProbs-WPGMA is doing better up to this point. Afterwards, the curve is decreasing, which means GLProbs-UPGMA is doing better. Therefore, as default, PnpProbs decides that a family has low discrepancy if its *σ*(PID) is smaller than 11.5 %, and uses the WPGMA method to construct its guide tree.

### Non-progressive alignment for distantly related sequences

**Fig. 3:**
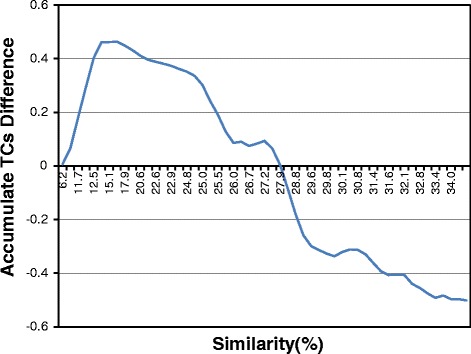
Accumulated TC score difference between PicXAA-AD and GLProbs

Recall that PnpProbs uses a non-progressive method to generate the MSA for distantly related sequences. To get more insight into the relative strength of the progressive and non-progressive methods, we have compared the performance of GLProbs with that of another MSA tool, PicXAA, which uses the nonprogessive sequence annealing method. To make the comparison more meaningful, we have modified the first stage of PicXAA so that it uses the same adaptive approach as GLProbs for generating substitution scores. We call the modified tool PicXAA-AD. Figure 3 shows the accumulated TC score difference between PicXAA-AD and GLProbs for aligning families in the three benchmark databases, namely SABmark, OXBench and BAliBASE. Note that the accumulated differences is increasing until the point around 18 %, and then is decreasing afterwards. This means that the non-progressive tool PicXAA-AD is doing better when the similarity of the input is less than 18 %, and the progressive tool GLProbs is doing better for the other inputs.

### The algorithm of PnpProbs

Given an input family of sequences, PnpProbs constructs its MSA as follows.

1. Calculate the percent identity (PID) for every pair of sequences, and compute the average *avg*(PID) and standard deviation *σ*(PID).
2. Use the *avg*(PID) to determine proper pair-Hidden Markov model(s) to compute the posterior probabilities.
3. Transform the posterior probabilities for consistency and use them as substitution scores.
4. Based on *avg*(PID) to determine which alignment approach to use:
  If *avg*(PID) <18 *%* (this is the distantly related sequences case, and we use the non-progressive sequence annealing technique to get the MSA)
  - Sort the probabilities *P*(*x*_*i*_,*y*_*j*_) in descending order.
  - Construct an acyclic graph with the same-column sets as its nodes, and insert the character pairs (*x*_*i*_,*y*_*j*_) to the graph iteratively according to the sort probabilities.
  - Topologically sort the graph, and from it constructs the MSA.
  If *avg*(PID) ≥ 18 % (this is the normally related sequences case.)
  - Compute the distance matrix for every pair sequences.
  - Determine the guide tree construction method based on some threshold *τ* on the standard deviation *σ*(PID) of the PIDs, whose default value is 11.5 *%* as suggested by our study in Section “Construct better guide trees for normally related sequences”: If *σ*(PID) <*τ*, use the WPGMA method to construct the guide tree; otherwise, use the UPGMA method
  - Based on the constructed guide tree, perform the profile-to-profile alignments to construct the MSA.
5. Refine the MSA given in the previous step as follows: we iteratively divide the MSA into two groups by randomly assign each sequence one of them, and we re-align these two groups using standard profile-profile alignment method to see if any improvement can be made. We stop when either (i) we have made 2*N* iterations and still cannot make any improvement, or (ii) we have made 4*N* iterations. Here, *N* is the number of input sequences.

## Results

To evaluate the performance of PnpProbs, we have compared it with thirteen other leading multiple sequence alignment tools on three popular benchmark databases. PnpProbs has the best performance in almost all cases, and it achieves significant improvements over the other tools on distantly related sequences. We have also studied its practicability by using it for phylogenetic analysis.

### Benchmark comparison

We have compared PnpProbs with the following multiple sequence alignment tools, ten of them use the progressive method: ClustalW 2.1, T-Coffee 9.03, MAFFT 7.031, MUSCLE 3.8.31, ProbCons 1.12, CONTRAlign 2.01, Probalign 1.4, MSAProbs 0.9.7, Clustal *Ω* 1.1.0, GLProbs, and three of them use the non-progressive method: Align-m 2.3, PicXAA, DIALIGN. We used these tools to align families of sequences obtained from the three benchmark alignment databases, namely OXBench 1.3, SABmark 1.65 and BAliBASE 3.0. To measure the accuracy of their alignments, we used the sum-of-pairs score (SP) and the total-column score (TC), which were commonly used in previous studies [2, 10, 11, 13, 15].

**Fig. 4:**
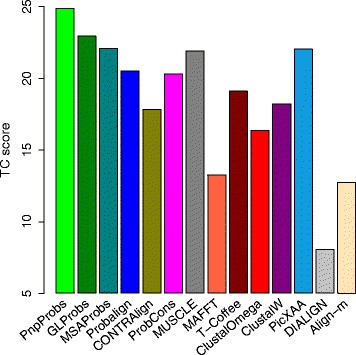
Mean TC score on OXBench (0–20 % similarity)

Table 1 compares the performance of the tools on OXBench. It is divided into four categories according to the similarities of the input families. For example, the category “ALL(0–100 %)” show the average SP and TC scores over all the input families used in the test, and the category “(0–20 %)” are for families with similarities between 0 and 20 %. Notice that PnpProbs achieved the overall highest SP and TC scores, and has big improvement for distantly related sequences. For example, Fig. 4 shows that for the category (0–20 %), PnpProbs achieved improvements over ClustalW by 36.5 %, over PicXAA by 12.9 % and over GLProbs by 8.4 %.

**Table 1:** Average SP and TC scores on OXBench

	ALL (0–100 %)		0 %–20 %		20 %–50 %		50 %–100 %		Time
	SP	TC	SP	TC	SP	TC	SP	TC	mm:ss
PNPProbs	**90.41**	**82.23**	**48.98**	**24.88**	83.47 ^∗^	**68.79**	**98.05**	**95.18**	2:58
GLProbs	90.38 ^∗^	82.14 ^∗^	47.29 ^∗^	22.95 ^∗^	**83.48**	68.65 ^∗^	**98.05**	**95.18**	3:15
MSAProbs	90.07	81.75	44.83	22.08	82.77	67.74	98.01	95.08	4:04
Probalign	89.97	81.68	43.58	20.51	82.53	67.46	**98.05**	**95.18**	2:10
CONTRAlign	89.34	79.87	44.76	17.83	81.56	64.75	97.55	94.10	10:19
ProbCons	89.68	80.86	44.15	20.30	82.06	66.33	97.84	94.61	1:48
MUSCLE	89.50	80.67	45.64	21.90	81.75	66.15	97.63	94.28	0:19
MAFFT	88.00	77.96	37.82	13.27	78.99	60.86	97.41	93.68	0:19
T-Coffee	89.52	80.50	43.99	19.11	81.82	65.85	97.75	94.38	15:05
Clustal *Ω*	88.91	79.99	39.09	16.38	80.71	64.49	97.76	94.58	0:12
ClustalW	89.43	80.16	42.94	18.23	81.67	65.01	97.76	94.40	0:22
PicXAA	89.64	80.74	45.11	22.04	81.86	65.91	97.84	94.55	4:26
DIALIGN	83.97	72.41	26.03	8.07	72.67	52.57	95.21	89.54	3:17
Align-m	86.95	76.06	28.36	12.74	76.35	57.54	96.95	92.60	21:14

The table shows the average SP and average TC score (multiplied by 100). The best and second best results in each column are marked in bold and with *, respectively. The last column shows the running time using a single CPU thread. Note that we use default parameters for all tools

Table 2 shows the average SP and TC scores for SABmark 1.65. The Twilight Zone contains sequences with less than or equal to 25 % similarity, and the Superfamily contains sequences with similarity mostly between 20–50 % similarity. Table 3 shows the average SP and TC scores for BAliBASE 3.0. RV11 contains distantly related sequences (with less than 20 % similarity) and RV12 contains medium to divergent sequences with similarities from 20 to 40 %. For these two benchmark databases, PnpProbs achieved the highest scores in most tests. Again, its improvement was more significant for distantly related sequences, i.e., Twilight Zone and RV11 subsets.

**Table 2:** Average SP and TC scores on SABmark

	ALL		Twilight Zone		Superfamily		Time
	SP	TC	SP	TC	SP	TC	mm:ss
PnpProbs	61.37 ^∗^	**41.70**	**44.40**	**24.80**	67.19 ^∗^	**47.49**	3:00
GLProbs	**61.42**	41.36 ^∗^	44.35 ^∗^	24.30 ^∗^	**67.27**	47.21 ^∗^	3:20
MSAProbs	60.27	40.02	42.97	22.88	66.20	45.90	1:58
Probalign	59.53	38.63	42.42	22.64	65.39	44.11	1:01
CONTRAlign	57.45	35.59	39.01	17.69	63.77	41.73	4:56
ProbCons	59.69	39.17	42.81	22.78	65.47	44.79	1:12
MUSCLE	54.51	33.47	34.69	16.96	61.29	39.13	0:46
MAFFT	52.63	32.57	31.72	15.17	59.79	38.53	0:22
T-Coffee	59.14	39.53	41.66	23.29	65.13	45.10	4:36
Clustal *Ω*	55.02	35.47	35.55	18.10	61.69	41.42	0:18
ClustalW	51.92	31.37	31.45	15.09	58.93	36.95	0:14
PicXAA	59.37	39.11	41.05	21.51	65.65	45.14	3:29
DIALIGN	47.09	27.11	27.85	12.73	53.69	32.05	1:03
Align-m	46.19	31.07	25.72	16.28	53.21	36.14	5:32

The best and second best results in each column are marked in bold and with *, respectively

**Table 3:** Average SP and TC scores on BAliBASE

	ALL		RV11		RV12		Time
	SP	TC	SP	TC	SP	TC	mm:ss
PnpProbs	82.80 ^∗^	**68.00**	68.91	**45.73**	94.79 ^∗^	87.23 ^∗^	3:22
GLProbs	**83.20**	67.59 ^∗^	**69.72**	44.68	**94.84**	**87.38**	4:05
MSAProbs	82.35	66.83	68.13	44.02	94.63	86.52	3:02
Probalign	82.53	67.27	69.50 ^∗^	45.34 ^∗^	94.63	86.20	1:47
CONTRAlign	77.59	58.10	61.78	35.60	91.23	77.52	6:37
ProbCons	81.55	65.22	66.99	41.68	94.12	85.54	1:41
MUSCLE	75.60	58.27	57.15	32.06	91.53	80.89	0:37
MAFFT	72.46	52.58	52.96	26.19	89.30	75.38	0:14
T-Coffee	80.82	64.93	65.63	41.36	93.94	85.29	5:18
Clustal *Ω*	75.96	59.38	59.01	36.21	90.60	79.38	0:21
ClustalW	69.63	49.21	50.06	22.99	86.52	71.84	0:21
PicXAA	81.33	66.08	66.56	44.06	93.47	84.19	3:26
DIALIGN	68.63	48.22	49.72	26.81	84.18	65.81	1:34
Align-m	71.45	56.04	51.88	33.06	88.36	75.88	7:09

The best and second best results in each column are marked in bold and with *, respectively

**Fig. 5:**
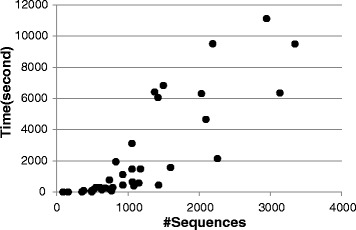
Running time of PnpProbs

For the efficiency of PnpProbs, we note from the last column of Tables 1, 2 and 3 that even using a single CPU thread, the running time of PnpProbs is comparable to most other tools. Moreover, it is straightforward to “parallelize” Step 1, 2, 3, 4a, 4i, 4iii and 5 of the algorithm of PnpProbs, and thus we can speedup PnpProbs’ execution easily by using multiple-cores CPUs. Figure 5 shows PnpProbs’ speed when running on a platform of six i7-3930k dual-cores with 64G RAM for inputs with different number of sequences. We note that PnpProbs takes an average of half an hour to align 1000 sequences.

### Phylogenetic analysis

To compare the practicability of PnpProbs with other existing tools, we have used it, as well as five other MSA tools, namely GLProbs, MSAProbs, PicXAA, MUSCLE and ClustalW, to construct phylogenetic trees. Given a set of sequences, we first used the six MSA tools to construct six MSAs, and used them as input to the Maximum Parsimony method [24] to infer six *hypothesized* phylogenetic trees. Then, for each of these hypothesized trees, we calculated the Robinson-Foulds(RF) distance [25] between the tree and the model phylogenetic tree; the smaller the distance, the closer the two trees, and hence the better the corresponding MSA. Our tests used input sequences chosen from two benchmark databases, namely Yule-Harding tree simulated data [17] and SABmark empirical data [5].

#### Simulated data

**Fig. 6:**
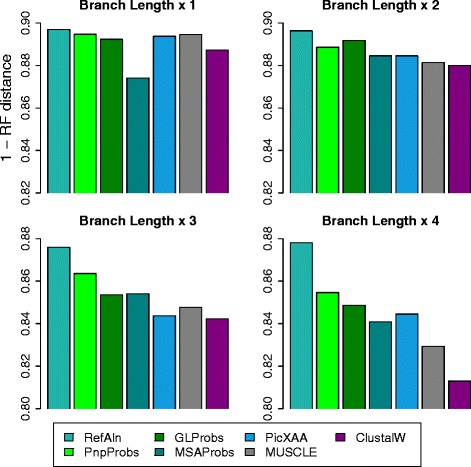
Similarity between hypothesized trees and model trees for simulated data

Figure 6 shows the results for inputs chosen from the Yule-Harding tree simulated database, which contains, for every family of sequences, a reference tree and a reference alignment for the family. We used the provided reference trees as the model trees to calculate the RF distance. We also use the reference alignment given in the database to construct a phylogenetic tree, and we refer this tree as RefAln.

**Fig. 7:**
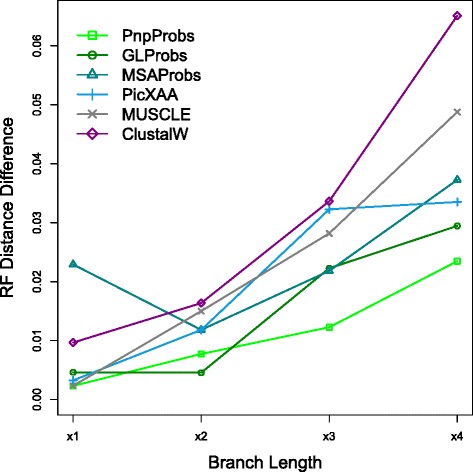
RF distance difference between RefAln and other hypothesized trees

Note that the Yule-Harding database is divided into four categories according to the simulated branch length diameter, and the larger the branch length diameter, the more divergent the phylogeny. Figure 6 uses 1−RF distance (i.e., 1 minus the RF distance) as the score for measuring the similarity of two trees. Note from the figure that in most cases, the hypothesized trees derived from PnpProbs’ alignments achieve scores higher than that of the other tools, and we can argue that the alignments of PnpProbs are better. Furthermore, as shown in Fig. 7, the RF distance differences between RefAln and the other hypothesized trees become larger when the phylogenies are more divergent (i.e., with larger branch length). However, the differences for PnpProbs increase mildly and are smaller than those of the five other MSA tools.

#### Empirical data

**Fig. 8:**
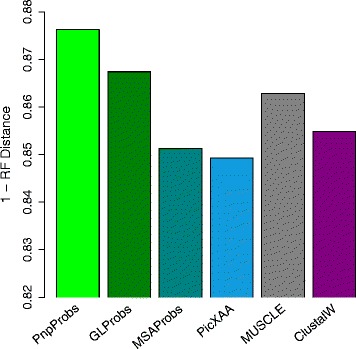
Similarity between hypothesized trees and model Trees for empirical data

Figure 8 shows the result for inputs obtained from the SABmark empirical database, which contains, for every family of sequences, a reference alignment. However, the database has no reference trees; thus we used RefAln as the model tree to compute the RF distance. We note that the hypothesized trees derived from PnpProbs’ alignments have the highest accuracy.

## Discussion and Conclusions

Our MSA tool PnpProbs aims at combining the strength of progressive and non-progressive methods for multiple sequence alignment; it uses progressive method for normally related sequences, and uses non-progressive method for distantly related ones. In [1], we proposed to use the average percent identity to estimate the similarity of a family of sequences, and in this paper, we proposed to use the standard deviation of the percentage identity to estimate the discrepancy of a sequence family. For normally related sequences, PnpProbs uses different methods to construct guide trees depending on the discrepancy of the family. Our experimental results showed that PnpProbs has the best TC scores in all but one test. We have also evaluated PnpProbs’ practicability, and our results suggested that PnpProbs will be a useful tool for downstream phylogenetic analysis.

For possible future research direction, we note that most of the MSA tools try a certain range of sizes of components to assemble multiple sequence alignment. For example, the progressive alignment method uses big components of sequence profiles, and the non-progressive sequence annealing technique uses small components, e.g., alignable columns or residue pairs. A natural research direction is to consider multiple sizes of decomposed components in one algorithm to build up the MSA such that families of sequences with long conserved regions apply large components and those with small conserved patterns use small components.
